# Hospital Course of a Man With Viral Pneumonia Caused by COVID-19

**DOI:** 10.7759/cureus.9261

**Published:** 2020-07-18

**Authors:** Matthew Solomon, Latha Ganti

**Affiliations:** 1 Emergency Medicine, Brown University, Providence, USA; 2 Emergency Medicine, Envision Physician Services, Nashville, USA; 3 Emergency Medicine, University of Central Florida College of Medicine/Hospital Corporation of America Graduate Medical Education Consortium of Greater Orlando, Orlando, USA; 4 Emergency Medical Services, Polk County Fire Rescue, Bartow, USA

**Keywords:** covid-19, pneumonia

## Abstract

The authors present a case of a man with pneumonia caused by COVID-19. There is currently no FDA-approved medical treatment or vaccine for COVID-19, so a variety of drugs and medicinal therapies have been repurposed for use in hospital settings and clinical studies while the medical community waits for a medication to be approved and standardized. Pneumonia is a common outcome of infection with severe acute respiratory syndrome coronavirus 2 (SARS-CoV-2), so cases of it are rapidly spreading around the world as the novel coronavirus continues to spread.

## Introduction

COVID-19 is an infectious disease caused by a novel coronavirus (one of seven) known as severe acute respiratory syndrome coronavirus 2 (SARS-CoV-2), which, like other coronaviruses, is thought to be zoonotic. The disease was first reported in 2019 in Wuhan, China and has since been deemed a pandemic, currently causing significant deaths, social readjustment, and economic loss [1]. COVID-19 is known to cause respiratory illness and can be identified in patients with the symptoms of fever, shortness of breath, fatigue, cough, and malaise. Although many have reported to be asymptomatic, the disease is particularly known to severely affect the elderly and those with co-morbidities and immunodeficiency disorders. In the latter populations, there have been relatively more reports of pneumonia and acute respiratory distress syndrome (ARDS) [2]. There are currently no approved or definitive treatments or vaccines for the virus; however, several drugs have been repurposed. Remdesivir, originally designed for the Ebola virus, is a proven broad-range antiviral agent against several RNA viruses [3]. Some reports have shown the efficacy of a hydroxychloroquine and azithromycin treatment in a hospital setting for pneumonia induced by COVID-19, although recently disputed by the World Health Organization (WHO) [4]. 

## Case presentation

**Figure 1:**
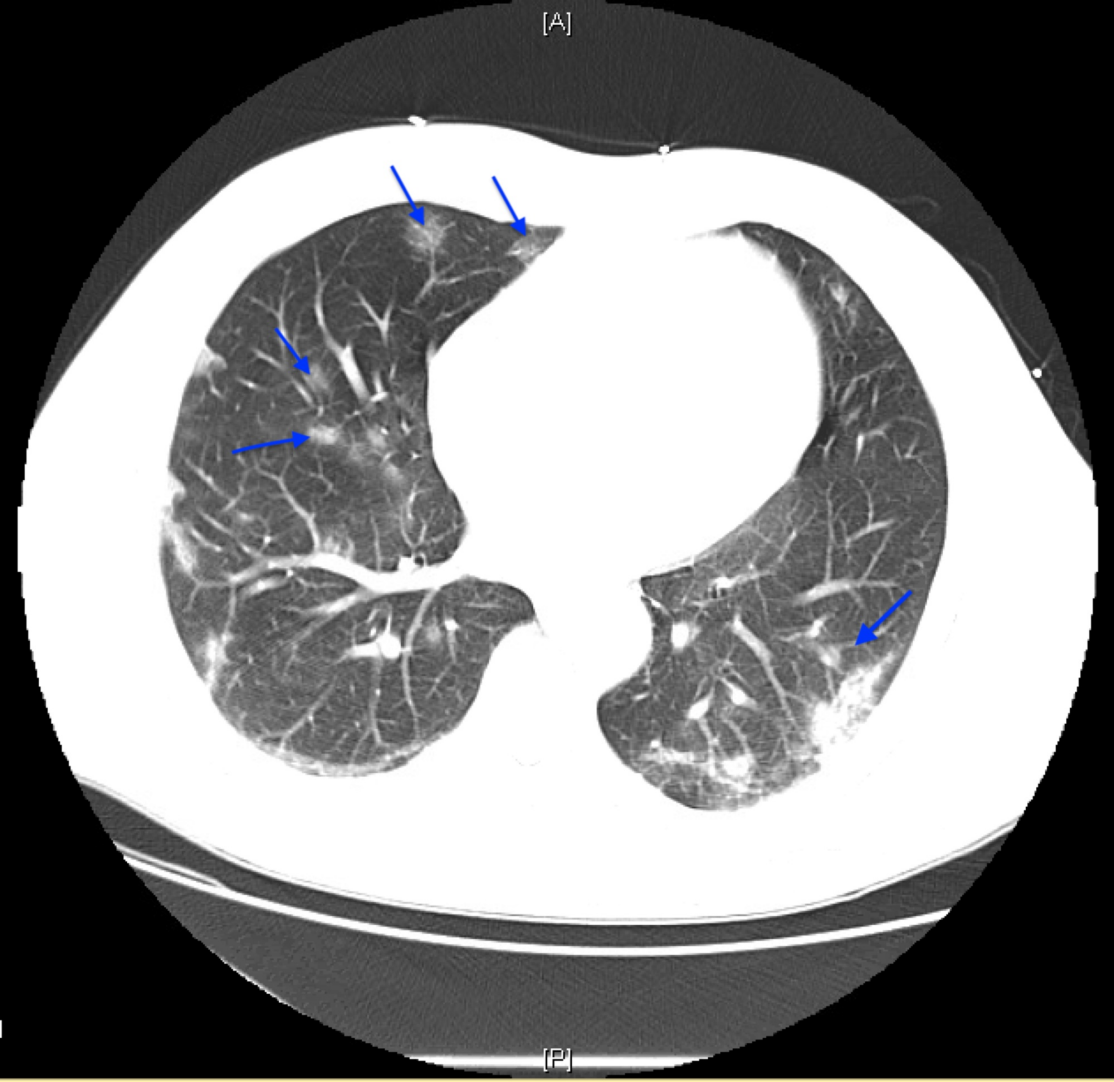
Non-contrast chest CT demonstrating patchy subpleural multifocal ground-glass opacities involving the bilateral upper and lower lobes (blue arrows).

A 61-year-old male with a past medical history of hypertension came to the emergency department (ED) with a chief complaint of cough and shortness of breath (SOB). One week prior, he had been to the ED with cough and SOB and was discharged home with azithromycin. The patient’s symptoms progressively worsened since his first ED visit, and now included associated diarrhea. Furthermore, the patient stated that nothing had made his symptoms better. The patient denied any sick contacts. He denied headache, vision changes, dizziness, chest pain, abdominal pain, nausea or vomiting, constipation, numbness, weakness, or headache. His vital signs included temperature 103.2⁰F, pulse 91 beats per minute, blood pressure 141/79 mmHg, and respiratory rate 22 breaths per minute. He had an oxygen saturation of 95% on room air which dropped into the low 90s with talking. The nurses reported further drop in oxygen saturation with ambulation. The sepsis protocol was implemented, including antibiotics. Given his history of hypertension, cardiac evaluation was also done. The patient was given 500 mg azithromycin and 1 g ceftriaxone intravenously. Additionally, a CT scan of his chest was done. The results showed patchy subpleural multifocal ground-glass opacities involving the bilateral upper and lower lobes as well as lingular and right middle lobe infiltrates (Figure 1).

There was no pleural effusion or thickening, and no pneumothorax. The patient was suspected to have a coronavirus as opposed to another cause of pulmonary infection. He was subsequently admitted and tested positive for the SARS-CoV-2 virus. The infectious disease specialist was consulted for evaluation and management. He also had hypoxemia, albeit stable, on exertion. At the time, he was on 3 L nasal cannula saturating at 98%. Subsequently, contact and drop isolation precautions were taken.

**Figure 2:**
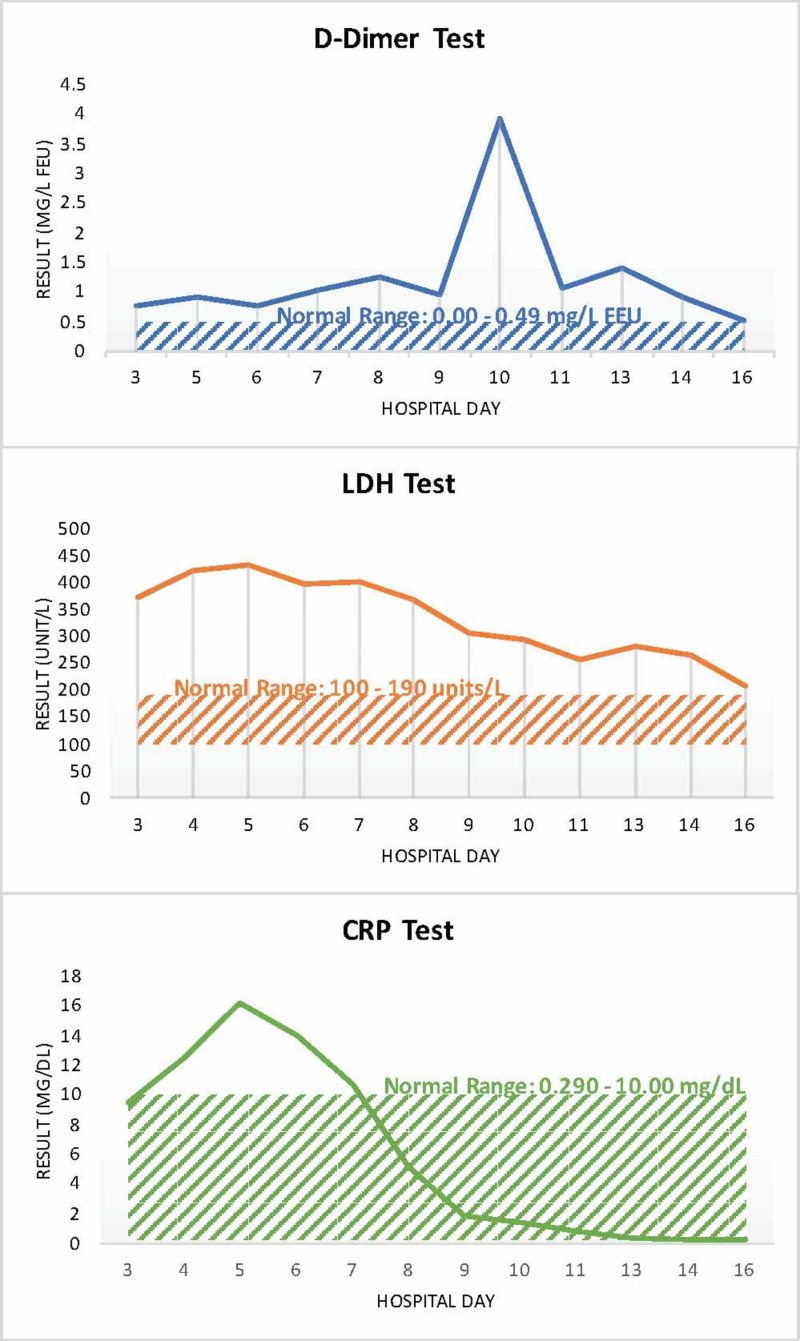
Graphs of patient's inflammatory markers over his hospital course. CRP, C-reactive protein; LDH, lactate dehydrogenase

On hospital day 3, the patient’s inflammatory markers began to be measured until discharge (Figure 2).

**Figure 3:**
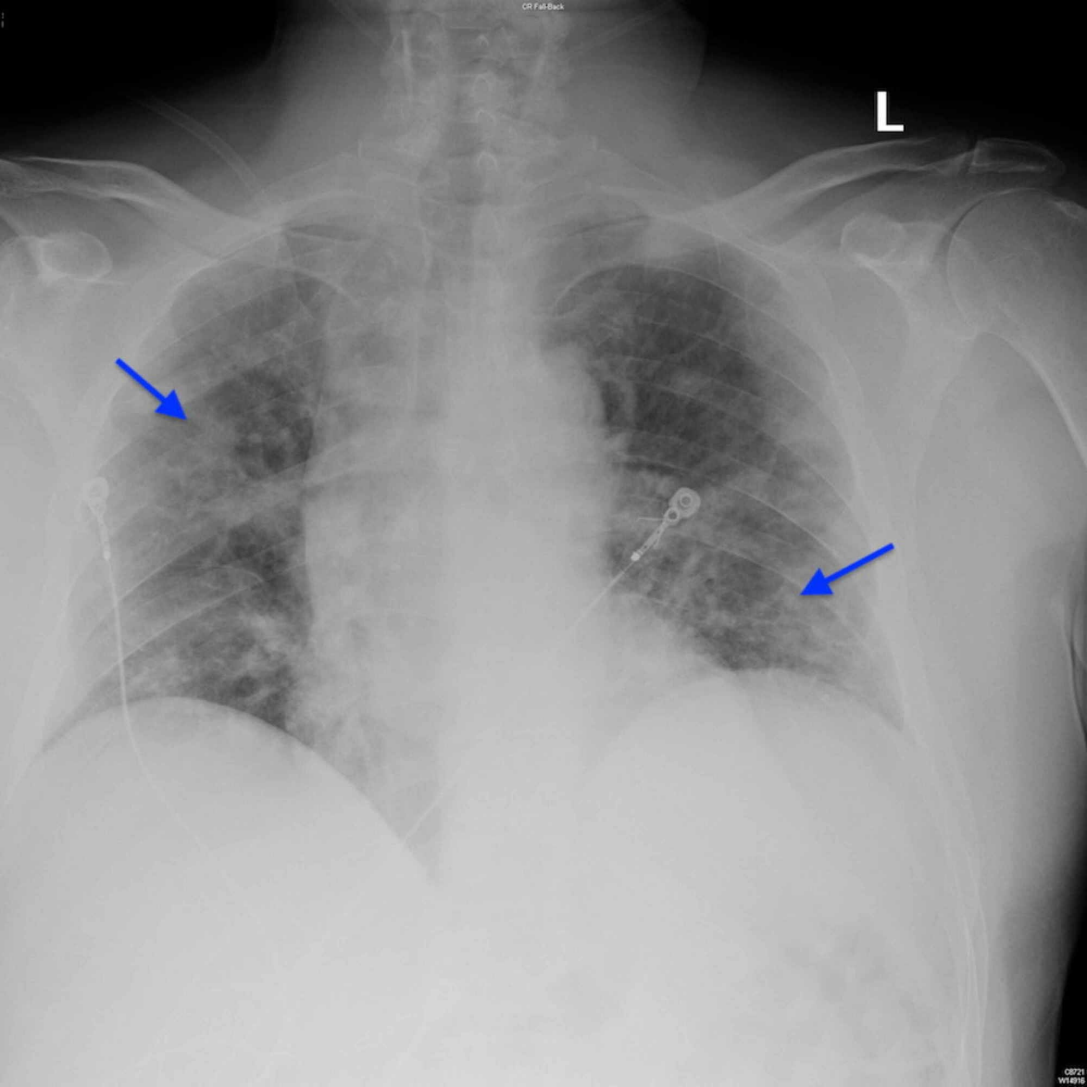
Chest radiographs demonstrating bilateral infiltrates (arrows).

At first, the patient’s inflammatory markers were relatively high: D-dimer mg/L FEU (fibrinogen equivalent units) was elevated at 0.75, lactate dehydrogenase (LDH) was elevated at 374 units/L, and C-reactive protein (CRP) was elevated at 9.44 mg/dL. The patient remained afebrile. He was given a five-day course of 400 mg hydrochloroquine to be administered orally and 2 g cefepime IV. Repeat X-ray of the patient showed worsening bilateral pneumonia (Figure 3).

On hospital day 5, the patient’s inflammatory markers worsened: D-dimer measured 0.92 mg/L FEU (normal = 0-0.49 mg/L FEU), LDH measured 434 unit/L (normal = 100-190 units/L), and CRP measured 16.2 mg/dL (normal = 0.290-10 mg/dL). However, the patient was not in respiratory distress. It was determined that the patient should continue the current treatment.

**Figure 4:**
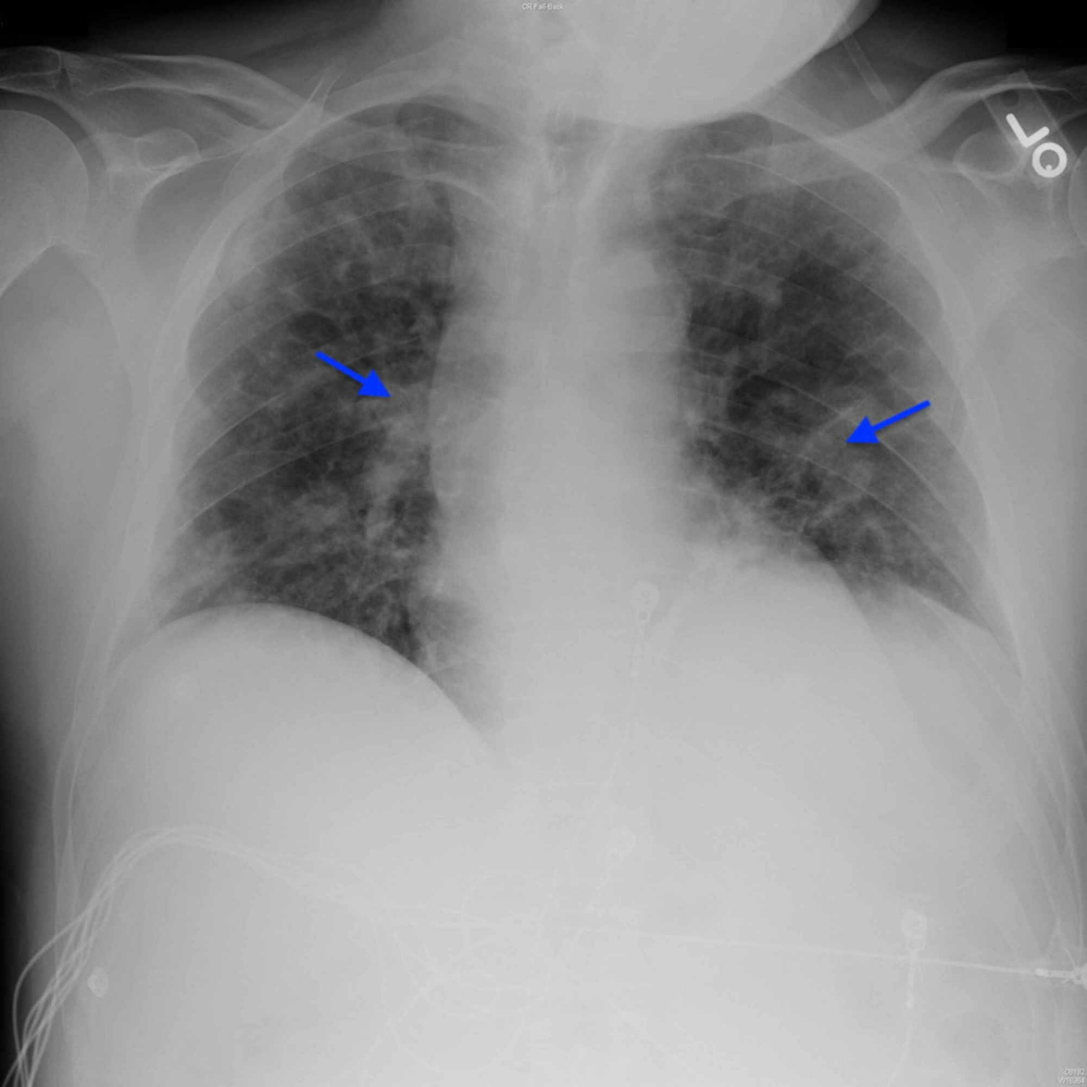
Chest radiograph on day 12 demonstrating stable patchy bilateral airspace opacities with superimposed interstitial thickening (arrows). Cardiomediastinal silhouette and osseous structures are unremarkable.

On hospital day 6, the patient was given a two-day course of 5 mg ivermectin to be administered orally and 400 mg tocilizumab IV. On hospital day 10, the patient was given convalescent plasma to combat COVID-19. On hospital day 12, a third and final chest x-ray demonstrated stable patchy bilateral airspace opacities with superimposed interstitial thickening (Figure 4).

After 16 days in the hospital, the patient had tested negative for SARS-CoV-2 and no longer experienced symptoms of viral pneumonia. In a stable condition, he was discharged home.

## Discussion

**Figure 5:**
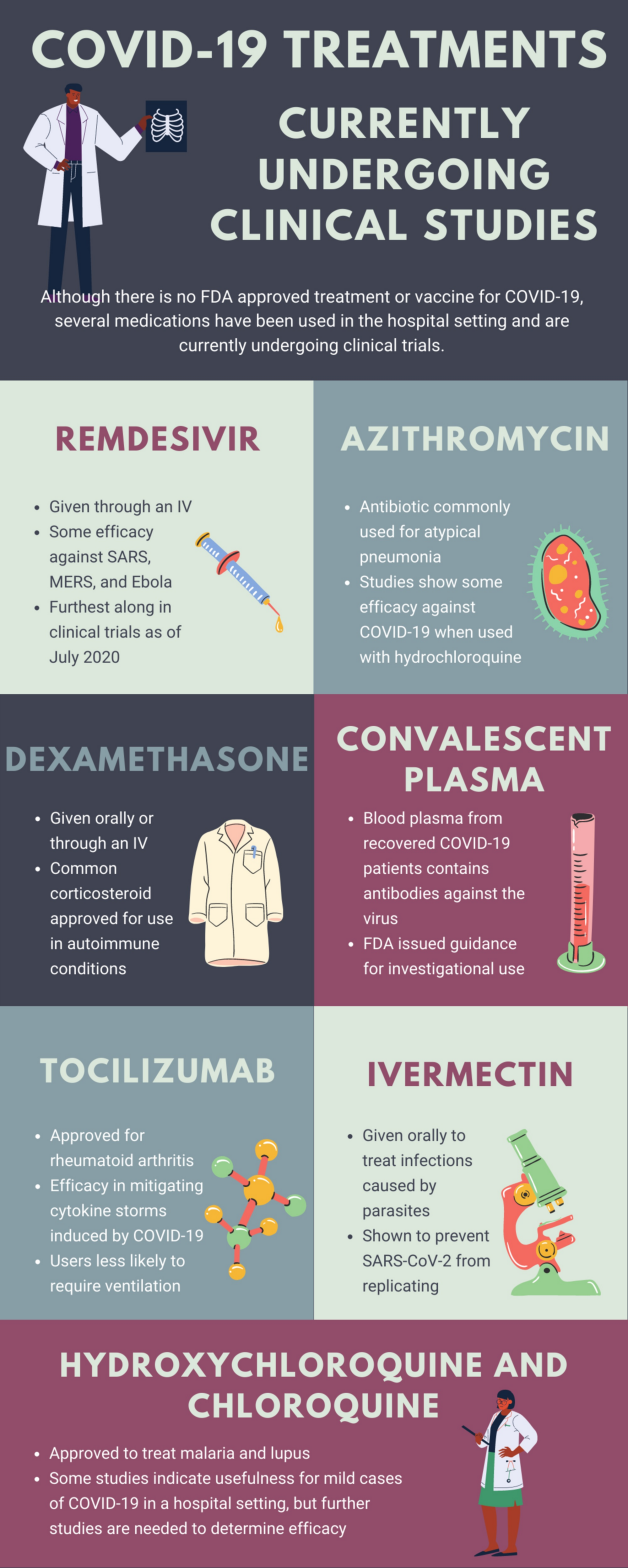
Infographic summarizing potential treatment options for COVID-19 as of July 2020. MERS, Middle East respiratory syndrome; SARS-CoV-2, severe acute respiratory syndrome coronavirus 2

Pneumonia is a common result of COVID-19 that is variably treated with a regimen of medications that are currently undergoing clinical studies (Figure 5) [5].

Although there is no FDA-approved medicinal therapy or vaccine for the novel coronavirus, this case illustrates that various drugs can be repurposed to treat COVID-19 patients on a temporary basis before an official medication is approved [6]. Before arriving at the emergency department, the patient received azithromycin, which was later seen to be ineffective by itself. However, when combined with other antibiotics or antiviral medications, it effectively treats COVID-19 patients. Specifically, in this case, a course of azithromycin paired with ceftriaxone had mildly positive effects. Azithromycin has shown greater efficacy when paired with hydroxychloroquine [4]. The patient’s inflammation (indicated by CRP and LDH counts) began to readily decrease midway through the five-day course of hydroxychloroquine and cefepime and at the start of the two-day course of ivermectin and tocilizumab (an immunosuppressive). Subsequently, the patient was given convalescent plasma. Although still being heavily researched, it has worked effectively in hospitals, notably successfully treating a centenarian with COVID-19 [7]. Other medicinal therapies have been successfully paired and repurposed to treat COVID-19 pneumonia, such as chloroquine and clarithromycin [8].

A recent study of COVID-19 pneumonia in Wuhan, China indicates that 11.7% of patients with COVID-19 pneumonia will worsen in a short period of time and ultimately die of multiple organ failure, particularly respiratory failure. Further, the mean age of patients was 57.6 years and 54.2% of the patients were men, roughly describing the patient in this case [9]. Because of the fatal threat of COVID-19 pneumonia to many in the global population, medical professionals must follow the current research on these repurposed drugs to provide their patients with the most effective treatments available.

## Conclusions

In the absence of a vaccine or a FDA-approved therapy for COVID-19, physicians will need to rely on case reports and stay abreast of clinical trials to decide what medication combination will be best for their individual patient. There are many existing drugs that are being re-purposed with varying degrees of success.

## References

[1] Ahn DG, Shin HJ, Kim MH (2020). Current status of epidemiology, diagnosis, therapeutics, and vaccines for novel coronavirus disease 2019 (COVID-19). J Microbiol Biotechnol.

[2] Singhal T (2020). A review of coronavirus disease-2019 (COVID-19). Indian J Pediatr.

[3] Zhai P, Ding Y, Wu X, Long J, Zhong Y, Li Y (2020). The epidemiology, diagnosis and treatment of COVID-19. Int J Antimicrob Agents.

[4] Ng KK, Ng MK, Zhyvotovska A, Singh S, Shevde K (2020). Acute respiratory failure secondary to COVID-19 viral pneumonia managed with hydroxychloroquine/azithromycin treatment. Cureus.

[5] (2020). The latest research on COVID-19 treatments and medications in the pipeline. https://www.goodrx.com/blog/coronavirus-treatments-on-the-way.

[6] Jan H, Faisal S, Khan A, Khan S, Usman H, Liaqat R, Shah SA (2020). COVID-19: review of epidemiology and potential treatments against 2019 novel coronavirus. Discoveries.

[7] Kong Y, Cai C, Ling L (2020). Successful treatment of a centenarian with coronavirus disease 2019 (COVID-19) using convalescent plasma [Epub ahead of print]. Transfus Apher Sci.

[8] Millán-Oñate J, Millan W, Mendoza LA, Sánchez CG, Fernandez-Suarez H, Bonilla-Aldana DK, Rodríguez-Morales AJ (2020). Successful recovery of COVID-19 pneumonia in a patient from Colombia after receiving chloroquine and clarithromycin. Ann Clin Microbiol Antimicrob.

[9] Du R-H, Liang L-R, Yang C-Q (2020). Predictors of mortality for patients with COVID-19 pneumonia caused by SARS-Cov-2: a prospective cohort study. Eur Respir J.

